# Oxidized Biomass and Its Usage as Adsorbent for Removal of Heavy Metal Ions from Aqueous Solutions

**DOI:** 10.3390/molecules27186119

**Published:** 2022-09-19

**Authors:** Bogdan-Constantin Condurache, Corneliu Cojocaru, Petrisor Samoila, Susana Felicia Cosmulescu, Georgeta Predeanu, Andra-Cristina Enache, Valeria Harabagiu

**Affiliations:** 1 Laboratory of Inorganic Polymers, “Petru Poni” Institute of Macromolecular Chemistry, 41A Grigore Ghica Voda Alley, 700487 Iasi, Romania; 2 COSFEL ACTUAL SRL, 95-97 Grivitei Street, 010705 Bucharest, Romania; 3 Research Center for Environmental Protection and Eco-Friendly Technologies (CPMTE), University POLITEHNICA of Bucharest, 1 Polizu Street, 011061 Bucharest, Romania

**Keywords:** oxidized wool, adsorption, heavy metal ions, kinetics, thermodynamics

## Abstract

Nowadays, very coarse wool fibers are considered waste biomass and are discarded at random or burned. Therefore, it is of actual interest to valorize coarse wool fibers as utile products. In this sense, we report herein an environmentally-friendly process for the preparation of a new material based on oxidized wool fibers and designed for efficient adsorption of heavy metals from wastewater. The morphology and the structure of the obtained product were characterized by scanning electron microscopy (SEM) coupled with an X-ray energy-dispersive module (EDX) and by Fourier-transform infrared spectroscopy (FTIR). Likewise, the performances of the oxidized wool fibers for the adsorption of heavy metal cations (Cu^2+^, Cd^2+^, Pb^2+^) from aqueous solutions were tested. The adsorption kinetics data were analyzed by applying the pseudo-first-order (PFO) and pseudo-second-order (PSO) kinetic models. The equilibrium of the adsorption process was investigated by using the Freundlich and Langmuir isotherm models. According to the Langmuir isotherms registered at 300 K, the maximum adsorption capacities of the oxidized wool were found to increase from Cu^2+^ (9.41 mg/g) and Cd^2+^ (10.42 mg/g) to Pb^2+^ (30.71 mg/g). Consequently, the removal efficiency of metal ions was found to vary in the range of 96.8–99.7%. The thermodynamic parameters (e.g., enthalpy, entropy, and Gibbs free energy) were calculated and discussed.

## 1. Introduction

Anthropogenic pollution with heavy metals is considered one of the major environmental issues, accelerated by the development of different human activities (e.g., industrial processes, vehicle emissions, agriculture, e-waste disposal, and waste treatments), which generated significant quantities of such persistent pollutants [1,2]. The industrial development has been conducted to the obvious economic growth of modern society, unfortunately it has also caused serious damage to the environment [3]. A cost–benefit analysis of the last decades disclosed a value of at least $200 billion in the United States alone that was spent to clean up hazardous waste using conventional technologies [4,5]. Generally, the term “heavy metals” is attributed to metals that have densities higher than 5 g/cm^3^, atomic weights greater than 50, and atomic numbers bigger than 20 [1,6]. On the other hand, heavy metals are the natural components of the earth’s crust. Consequently, the process of rock erosion, natural degradation of plants and animal waste, precipitation, and airborne particles accumulated in the atmosphere (from volcanic eruptions and fire smoke) are all contributing to environmental pollution [1].

Some heavy metals are essential in many structural and biochemical functions of plants (including their growth, e.g., Cu^2+^) [7,8]. By contrast, other heavy metals (i.e., Pb^2+^ and Cd^2+^) are non-essential and have no known biological function, being toxic to plants even at low concentrations [9]. The essential elements for plant, animal, or human nutrition are required in low concentrations and are known as “trace elements” or “micro-nutrients” [10,11]. Hence, many metallic cations reveal high toxicity even at low concentrations. These heavy metals are accumulated in living organisms causing various disorders and diseases [12].

The main techniques employed to reduce the metal content in polluted waters deal with chemical precipitation, ion exchange, membrane filtration, reverse osmosis, solvent extraction, electrolytic methods, and adsorption [12,13,14]. Many of these methods are limited by high operating costs and/or inefficiency in removing heavy metal ions, mainly at low concentrations [1,15].

Recently, based on their adsorption capacity for metals, natural fibrous materials have attracted the attention of scientists for the development of new types of adsorbents. For this purpose, wool has proved to be a relevant low-cost adsorbent for the removal of heavy metal ions from various polluting sources and the purification of contaminated water sources [16]. The adsorption of metals by natural fibers can be enhanced by their physical/chemical modification. Hence, the wool fibers were electron-beam-irradiated [17] or treated with sodium sulfide [18] and their metal cation adsorption performances were investigated. 

The present study continues our pursuit of aiming to turn the waste wool fibers of very coarse quality into high-value-added materials that can be employed as sorbents for environmental protection applications. In our previous work [19], coarse wool fibers were utilized to produce a magnetic composite that was efficiently used for oil spill sorption. The idea of the current work was to increase the content of superficial ionic groups of wool fibers, in order to increase their capacity for linking metal cations. Thus, the paper deals with wool fiber oxidation in an alkaline medium and with the investigation of the performances of the newly obtained material in the removal of heavy metal cations (Cu^2+^, Cd^2+^ and Pb^2+^) from aqueous solutions.

## 2. Results and Discussion

**Scheme 1:**
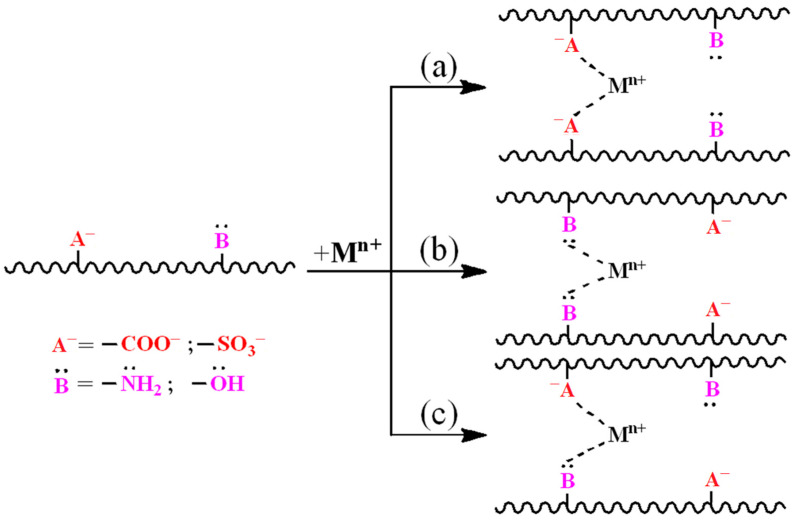
Representation of the possible mechanisms of adsorption of heavy metal ions onto the surface of oxidized wool fibers: (**a**) ionic interactions; (**b**) coordinative (donor–acceptor) interactions, and (**c**) mixed ionic/coordinating interactions.

The chemical composition of wool fibers is a complex one, with different functional carboxyl, amino, hydroxyl, disulfide and thiol groups that have relevant affinities for heavy metal ions [17,20]. The most prone metal ion binding sites are the carboxyl groups of aspartic and glutamic acid found in the structure of keratine [21,22]. These affinities rely on electrostatic attraction and donor/acceptor interactions between the metal cations and the surface of the material [17,23]. For instance, the carboxyl groups in their anionic form (–COO^−^) are able to attract positively charged metal cations by electrostatic forces (ionic bonds). In turn, the pair of non-participating electrons of the nitrogen in the amine groups (–NH_2_) or of the oxygen in the hydroxyl groups (–OH) may interact with metal cations present in the solution through coordinative (donor/acceptor) bonds [24]. Also, mixed electrostatic and coordinating wool–cation interactions could be envisaged, depending on the solution pH [21,24,25]. Scheme 1 shows a suggested scenario of the mechanism of adsorption of heavy metal ions on oxidized wool fibers.

Wool fibers were already proved as appropriate sorbents for heavy metal cations [20,23,26,27,28,29,30,31]. Analyzing the dedicated references, one may affirm that the cations’ sorption performances of the wool-based materials are strongly affected by their chemical composition and morphology, their specific surface, etc. To increase the superficial content of anionic groups of wool fibers, their oxidation was performed as discussed in the next section.

### 2.1. Oxidized Wool Fibers

To increase the surfaces functionality of the wool fibers, they were oxidized in the presence of a mixture of H_2_O_2_ and NH_4_OH, as mentioned in Section 3.2. Newly formed functional groups were expected to emerge as a result of the oxidation process. For example, herein we might expect the appearance of carboxyl and cysteine oxide groups generated by the oxidation of amide bonds from the chain backbone of wool keratin, respectively, of the disulfide cross-linking bridges.

**Figure 1:**
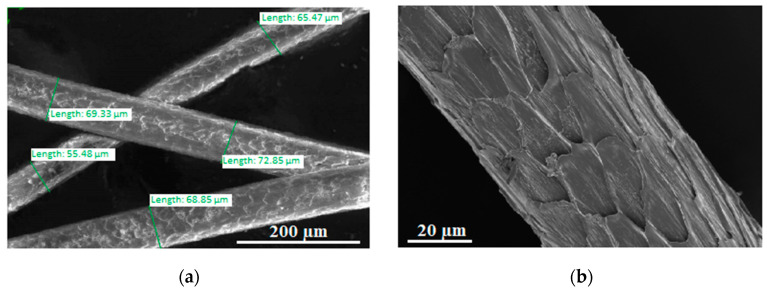

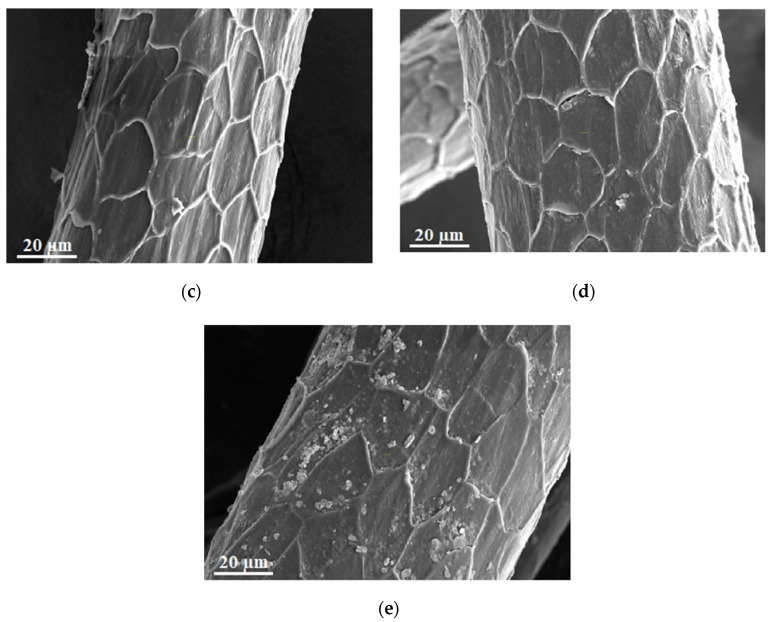
SEM images of: (**a**) pristine wool fibers, (**b**) oxidized wool fibers, (**c**,**d**,**e**) oxidized wool fibers loaded with Cu^2+^, Cd^2+^, respectively Pb^2+^.

**Figure 2:**
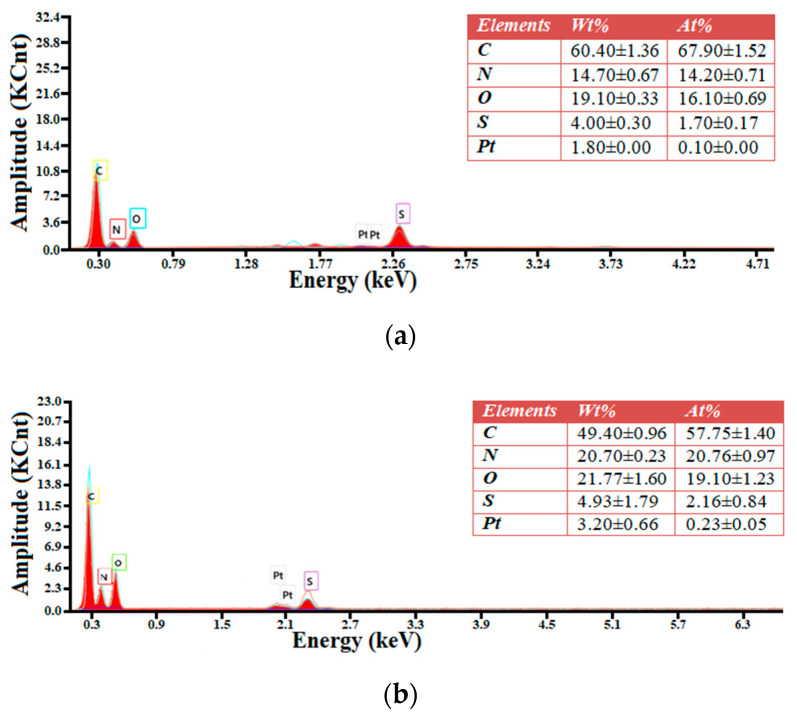

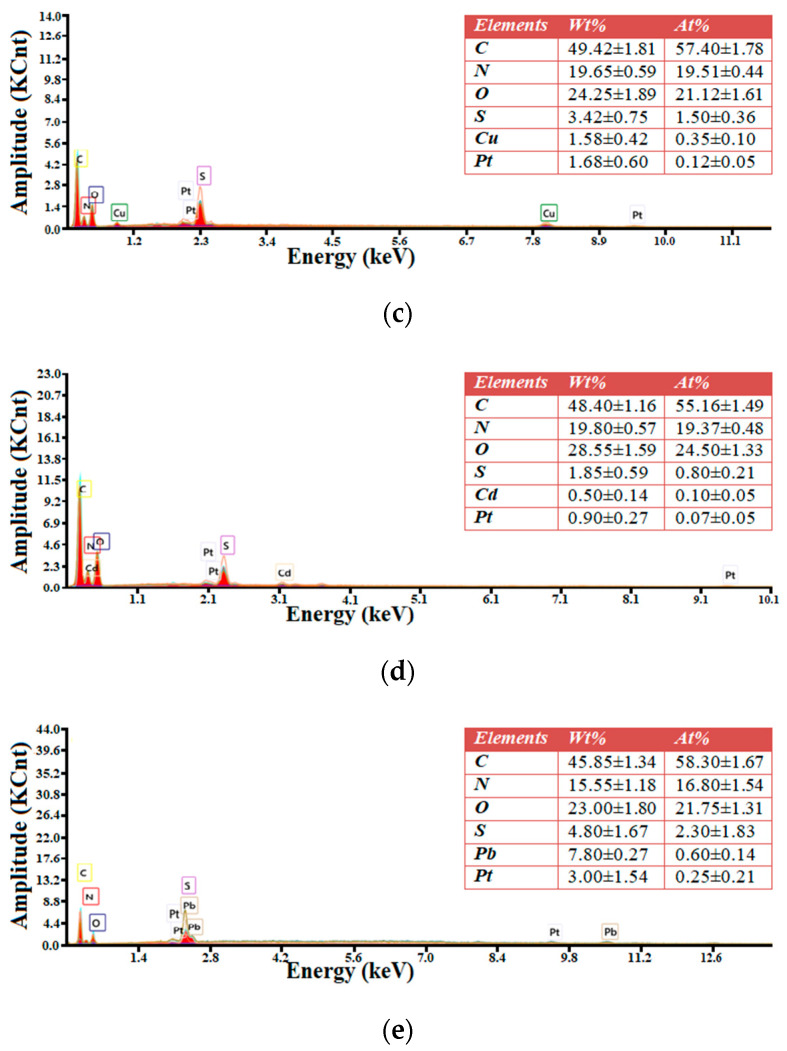
EDX spectra and the elemental composition (inset) of: (**a**) oxidized wool fibers, (**b**) oxidized wool fibers, (**c**,**d**,**e**) oxidized wool fibers loaded with Cu^2+^, Cd^2+^, respectively Pb^2+^.

**Figure 3:**
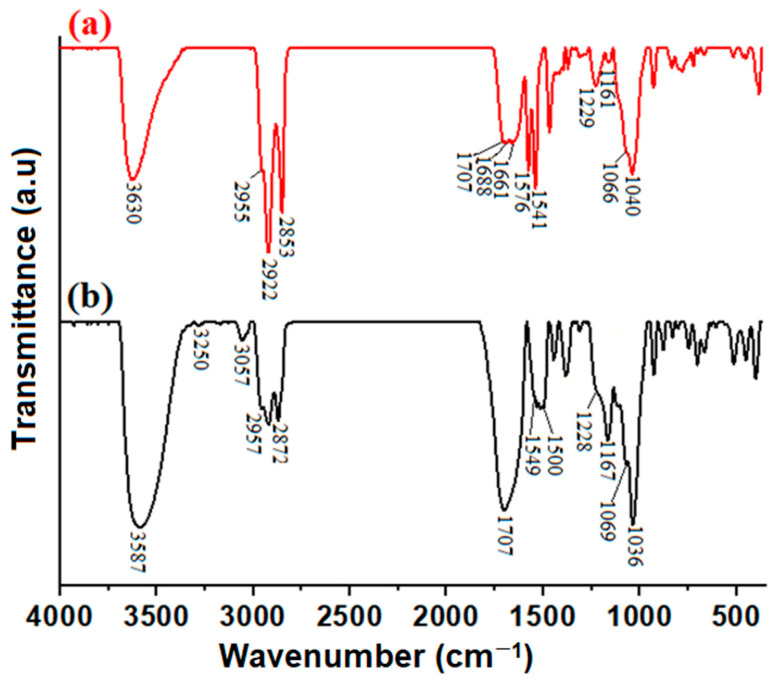
FTIR spectra of wool-based fibers: (**a**) pristine wool fibers; (**b**) oxidized wool fibers.

The surface morphology and the structural characteristics of pristine wool as compared with those of the oxidized wool fibers were investigated by using scanning electron microscopy (SEM) coupled with energy-dispersive X-ray (EDX) spectroscopy (Figure 1 and Figure 2, respectively) and FTIR analysis (Figure 3).

According to SEM images, pristine wool was found to be of very coarse grade [32] as the average fiber diameter is 66.0 ± 6.7 μm (Figure 1a). The fibers show a roughness emerging as a scale-like pattern (cuticle cells), which is characteristic of wool fibers [28]. This specific surface morphology does not change following oxidation (Figure 1b). The EDX spectra of both pristine (Figure 2a) and oxidized (Figure 2b) samples unveiled the presence of all expected chemical elements, i.e., C, O, N, S (Pt is due to the fiber metallization). As well known, EDX elemental analysis provides local element concentrations and depends on the depth from the surface the elements are counted. To have a better certitude on the fiber elemental composition, five different zones on the fibers were analyzed. The weight and atomic proportions reported in the inset tables (Figure 2) represent the average values. 

Hence, the EDX technique provides qualitative elemental analysis, which is surface-specific (i.e., the estimation has a local character). This could be the reason the obtained composition (C = 60.4%; N = 14.7%; O = 19.1%; S = 4.0%) of the pristine wool is somewhat different as compared to the averages values found for wool in the literature (C = 50%; N = 16–17%; O = 22–25%; S = 3–4%) [33]. Looking to the composition of pristine (Figure 2a) and oxidized wool fibers (Figure 2b), one may observe that both oxygen and nitrogen contents of the oxidized sample are higher as compared to the pristine one.

To further clarify the structural aspects, Figure 3 compares the specific IR absorptions of pristine wool and of the oxidized sample. The interpretation of the FTIR spectra of both materials was performed based on the information available in the literature [33,34,35,36,37,38,39,40]. As one may see from Figure 3a, the pristine wool shows the following characteristic absorptions: a large band centered at 3630 cm^−1^ (O–H groups and adsorbed water possibly superposed on amide A absorptions), bands located between 2955 and 2853 cm^−1^ (C–H vibrations), 1707 and 1688 cm^−1^ (COO- from aspartic and glutamic units), 1661 cm^−1^ (C=O, amide I vibration specific to secondary structure of wool keratin), 1576 and 1541 cm^−1^ (C–NH, amide II), 1229 cm^−1^ (amide III vibrations), 1161, 1066, and 1040 cm^−1^ (oxidized cystine groups, already present in the pristine sample). After oxidation (Figure 3b), the amide A (3250 cm^−1^), amide B (3057 cm^−1^) and amide III absorptions (1228 cm^−1^) become more visible, while the amide II vibrations move to the 1549–1500 cm^−1^ region. Moreover, the increase of the intensities of O–H (3587 cm^−1^), carboxylate overlapped on amide I (1707 cm^−1^) and of cysteine oxides bands (1167, 1069, 1036 cm^−1^) as compared to those of C–H groups (2957–2872 cm^−1^) is easily visible, confirming the higher content of oxygen in the oxidized sample evidenced by EDX analysis. Similar results were obtained when keratin was extracted by using H_2_O_2_ in alkaline media [36].

**Scheme 2:**
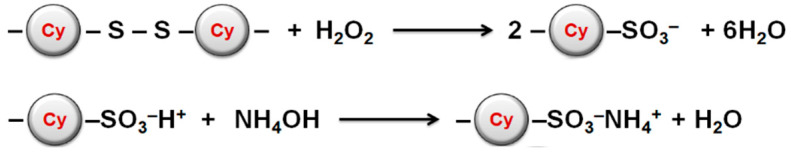
Reaction scheme for splitting of disulfide linkage (cystine) and neutralization of cysteic acid groups with ammonium hydroxide.

Thus, the increase of the oxygen and nitrogen contents in the oxidized sample could be mainly explained by the splitting of disulfide cystine linkage, respectively, by the neutralization of cysteic acid groups with ammonium hydroxide, according to the reactions represented in Scheme 2.

### 2.2. Adsorption/Desorption of Metal Cations on/from Wool Fibers

Once the superficial composition of the oxidized wool fibers was identified, one may propose a possible scenario of the mechanism of adsorption of the heavy metal cations on the oxidized wool fibers (Scheme 1). Many years ago, Friedman [21] demonstrated that the free carboxyl groups of aspartic and glutamic acid (available in the amorphous polypeptide sequences of wool), almost completely dissociated at pH 7 as well as cystine groups, seem to be the most prone to bind metal cations. Instead, the coordination binding of metal cations to amine and amide groups takes place in alkaline pH conditions [21,24].

The SEM images in Figure 1c–e show that the morphology of wool fibers was kept after the cation sorption, while the EDX elemental analysis identified the presence of the adsorbed Cu^2+^ (Figure 2c), Cd^2+^ (Figure 2d) and Pb^2+^ (Figure 2e) cations.

#### 2.2.1. Adsorption Kinetics

The adsorption kinetics of three representative heavy metal ions (Cu^2+^, Cd^2+^, Pb^2+^) onto the surface of the oxidized wool fibers was investigated for single-component systems (i.e., retention of each metal ion separately) and for a multi-component system (i.e., competitive retention of three metal ions simultaneously).

**Figure 4:**
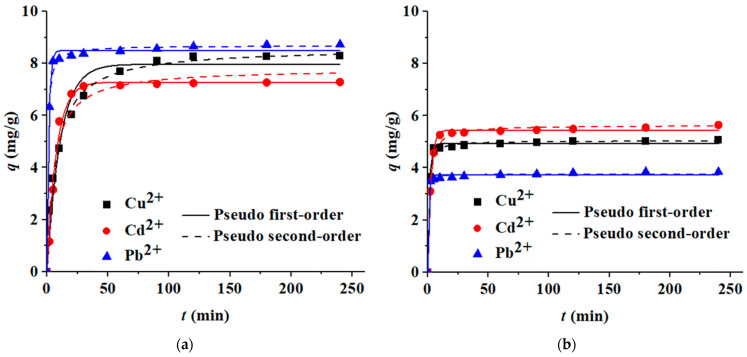
Adsorption kinetics of heavy metal cations (Cu^2+^, Cd^2+^, Pb^2+^) onto the solid surface of oxidized wool fibers: (**a**) adsorption kinetics for one-component systems (sorbent dose = 5 g/L; initial concentration of metal ion C_0_ = 50 mg/L; T = 300 K); (**b**) adsorption kinetics for competitive or multi-component system (sorbent dose = 5 g/L; initial concentration of each metal ion in the system was equal to 20 mg/L, summing up a total metal ions concentration of 60 mg/L; T = 300 K); solid lines represent predictions given by pseudo-first-order (PFO) kinetic model, and dash lines represent predictions given by pseudo-second-order (PSO) kinetic model.

Figure 4 illustrates the relationship between the adsorption capacity q_t_ (mg/g) and the contact time t (min) for the single-solute systems (Figure 4a) and for the competitive system (Figure 4b). The equilibrium adsorption capacities were found to be 8.73 mg/g (for retention of Pb^2+^), 8.30 mg/g (for retention of Cu^2+^), and 7.29 mg/g (for retention of Cd^2+^). For the multi-component system (Figure 4b) the results revealed the best selectivity for Cd^2+^ ions and the lowest selectivity for Pb^2+^ ions. By comparison with the single component systems, the adsorption capacities of oxidized wool strongly decreased when all the studied pollutant cations were mixed together. At equilibrium, the sum of the three maximum adsorption capacities was equal to 24.3 mg/g when summing up the adsorption capacities for the individual cation solutions. In turn, the same totaling value was found to be 14.6 mg/g, when summing up the adsorption capacities for the multi-component system. This discrepancy might be attributed to the fact that these two situations are not thermodynamically equivalent, that is, the equilibria are established in different ways. In this sense, ionic strength plays an important role. For instance, the salt source for Cu^2+^ ions was copper sulfate (CuSO_4_ × 5H_2_O). Instead, for Pb^2+^ and Cd^2+^ ions, their nitrate salts were employed, i.e., (Pb(NO_3_)_2_ and Cd(NO_3_)_2_ × 4H_2_O). Hence, for the individual systems, the following ionic strengths were calculated: (1) I1 = 0.80 mM for CuSO_4_, (2) I2 = 0.49 mM for Cd(NO_3_)_2_, and (3) I3 = 0.45 mM for Pb(NO_3_)_2_. Whereas, for the multi-component system (Cu(II)+Cd(II)+Pb(II)), the ascertained ionic strength was equal to I = 0.70 mM. Notice that this value is lower than the value of the ionic strength for the Cu^2+^ individual system. Thus, these evident discrepancies between ionic strengths of different adsorption systems may contribute to the discrepancy between the sum of the adsorption capacities for the multi-component system and individual systems. Therefore, the maximum capacity values were reduced in the multi-component system compared to individual systems as follows: by 56.01% for lead cations, 39.06% for copper cations and by 22.50% for cadmium cations, respectively. These findings are in close agreement with previous literature reports [41,42] and are usually attributed to the significant competition between the studied cations on the adsorption sites of the oxidized wool [41]. According to the literature, there are numerous factors that can be considered for ordering the cations adsorption capacities in multi-component systems, such as: covalent index, ionic charge, ionic radii or hydrated ionic radii, electronegativity, among others [41,42,43]. In our study, adsorption capacities follow the order: Cd^2+^ > Cu^2+^ > Pb^2+^ for the multi-component system. This result indicates that the order is reversely correlated with the hydrated ionic radii of the cations (4.01 Å for lead, 4.19 Å for copper and 4.26 Å for cadmium [43]). In fact, one may suppose that, once the bulkiest hydrated cation Cd^2+^ were adsorbed on the oxidized wool surface they are hindering the access of the other cations to the active adsorption sites. This fact resulted in the stronger decrease of the adsorption capacity values for Pb^2+^ and Cu^2+^ individually and in the premature exhaustion of the adsorbent. In addition, the slower diffusion of Pb^2+^ might be also associated with a greater atomic radius (1.80 Å) of this cation, compared to Cd (1.55 Å) and Cu (1.35 Å) atoms [44]. Once the adsorption equilibrium in the multi-component system has been established, it might be assumed that the subsequent exchange between these three cations is minimized. This might be deduced from experimental observations regarding desorption in distilled water, which showed insignificant desorption of heavy metal cations in water eluent (as further discussed in Section 2.2.4). Generally, the adsorption capacity gets higher with the increment of the contact time, until the stationary regime (equilibrium) is established. According to kinetic data reported in Figure 4, the adsorption equilibrium is generally established for contact time of more than 60 min. 

The experimental data were interpolated by means of the pseudo-first-order (PFO) and pseudo-second-order (PSO) model equations given by Equation (1) and Equation (2), respectively [45]:

(1) $$q_{t}={\;q}_{e}\;\left(1-e^{-k_{1}t}\right),$$

(2) $$q_{t}=\frac{k_{2}q_{e}^{2}\;t}{1+k_{2}q_{e}t}\;\;,$$

where q_t_ and q_e_ represent the adsorption capacities at time t and at equilibrium, respectively; k_1_ and k_2_ are constants for the equilibrium rate of the PFO and PSO adsorption. The parameters of kinetic models were calculated by the non-linear regression tool implemented in OriginPro software (version 9.9.0.225/SR1 2022). The concordance between observations and models was estimated by using the reduced χ-square statistic test ($\chi_{v}^{2}$) as the error function, which can be calculated according to Equation (3):

(3) $$\chi_{v}^{2}=\frac{1}{n-p}\overset{n}{\underset{i=1}{\sum }}\frac{{\left(q_{i}^{\left(\mathrm{obs}\right)}-q_{i}^{\left(\mathrm{calc}\right)}\right)}^{2}}{\sigma_{i}^{2}}\;,$$

where q^(obs)^ denotes the observed adsorption capacity (mg/g); q^(calc)^ is the calculated adsorption capacity (mg/g); i represents an integer index of summation; σ_i_^2^ is the variance (if no data replication, then σ_i_^2^ = 1); n and p represent the numbers of observation and fitted parameters, respectively, and the difference n – p gives the degree of freedom (v = n – p). Typically, the lower the error function is, the better the model prediction. As one can see from Figure 4 and by inspecting $\chi_{v}^{2}$ values (Table 1 and Table 2), both PFO and PSO kinetic models interpolate appropriately the experimental data. However, one model may be somewhat better than another one for a particular situation. For example, for the adsorption of Cu^2+^ ions in the single-component system, the PSO kinetic model gives a better prediction. Instead, Cu^2+^ adsorption in the competitive system is appropriately predicted by both PFO and PSO models.

#### 2.2.2. Adsorption Isotherms

**Figure 5:**
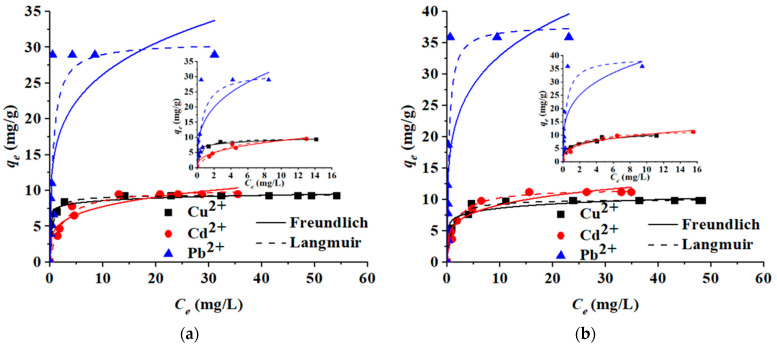
Adsorption isotherms of heavy metal cations (Cu^2+^, Cd^2+^, Pb^2+^) onto the solid surface of oxidized wool fibers: (**a**) adsorption isotherms recorded at 300 K; (**b**) adsorption isotherms recorded at 323 K; solid lines represent predictions given by the Freundlich model, and dash lines represent predictions given by the Langmuir model.

Isotherms describe the equilibrium between adsorption and desorption phenomena at a given temperature, representing the basis for the design of the adsorption system. The importance of adsorption isotherms relies on detailed information on the distribution of the adsorbate species (metal cations in this case) between the liquid phase and solid surface of the adsorbent at equilibrium and at different temperatures. In this study, the adsorption isotherms were explored at two different temperatures (300 K and 323 K), and the adsorption capacity was recorded at the contact time t = 240 min to ensure the attaining of the equilibrium. Figure 5 presents the adsorption isotherms describing the retention of heavy metal cations (Pb^2+^, Cd^2+^, Cu^2+^) onto the oxidized wool fibers at 300 K (Figure 5a) and 323 K (Figure 5b). As shown in Figure 5, as the equilibrium concentration C_e_ (mg/L) increases, the adsorption capacity q_e_ (mg/g) gets higher as well, attaining a stationary plateau for a given level of temperature. The equilibrium data revealed that the adsorption of Pb^2+^ cations is favored by the increase in temperature, while the adsorption of Cu^2+^ and Cd^2+^ cations is practically not so sensitive to temperature variation. This observation is analyzed in more detail in the next section dealing with the thermodynamics of the adsorption process. The experimental data were interpolated by using Freundlich and Langmuir isotherm equations (Equations (4) and (5), respectively), which are the most frequently used models in this sense [46,47,48,49,50]:

(4) $$q_{e}=K_{F}C_{e}^{1/n_{F}}\;,$$

(5) $$q_{e}=\frac{q_{m}K_{L}C_{e}}{1+K_{L}C_{e}},$$

where q_e_ and q_m_ represent the adsorption capacity at equilibrium and the maximum adsorption capacity, respectively, C_e_ is the metal concentration at equilibrium, K_F_ and K_L_ are the Freundlich and Langmuir isotherms constants, and 1/n_F_ is a variable heterogeneity factor (between 0 and 1). 

According to the Langmuir model, every adsorption site is energetically equivalent, and it can hold just one adsorbate molecule. Thus, the Langmuir approach only considers the formation of a monolayer of adsorbate onto the outer surface of the adsorbent. In turn, the Freundlich isotherm considers both the initial adsorption onto the heterogeneous surface as well as the condensation effect occurring due to adsorbate–adsorbate interaction [50]. Previous research has shown that the non-linear regression methods were more precise in estimating the model parameters compared to the linear regression techniques employed for the same purpose [45]. Hence, in this work, the non-linear regression method was applied with the aid of the OriginPro program in order to calculate the isotherm equations parameters. Also, the error function ($\chi_{v}^{2}$) was calculated to estimate the goodness-of-fit. Table 3 lists the calculated parameters of the isotherm models along with computed $\chi_{v}^{2}$ values. According to Figure 5 and Table 3, the Langmuir isotherm model provided better predictions than the Freundlich model. Hence, the Langmuir approach (i.e., monolayer adsorption) seems to represent better the equilibrium distribution of metal ions between the oxidized wool fibers and the liquid phase. However, both models for the adsorption isotherms for Pb^2+^ show important deviation from the experimental values at low equilibrium concentration (Figure 5) and the corresponding computed $\chi_{v}^{2}$ values are quite high (Table 3). The maximum adsorption capacities at 300 K were found to be 9.41, 10.42, and 30.71 mg/g for retention of Cu^2+^, Cd^2+^, and Pb^2+^ cations, respectively. These values were found to be substantially greater than the values registered for the adsorption capacities of pristine wool fibers (2.49, 3.87, and 8.66 mg/g for retention of Cu^2+^, Cd^2+^, and Pb^2+^ ions, respectively). At 323 K, the maximum adsorption capacities were somewhat higher (Table 3).

Further analysis of the Langmuir equation may allow the calculation of the dimensionless parameter (R_L_) for equilibrium, also known as the separation factor, which may be expressed by Equation (6) [46,50,51,52]:

(6) $$R_{L}=\frac{1}{1+K_{L}C_{0}}\;,$$

where C_0_ denotes the initial solute concentration, and K_L_–Langmuir constant. The calculation of the separation factor (R_L_) may offer information on whether the studied adsorption process is favorable or not. Generally, if the separation factor ranges between 0 and 1, then the studied adsorption process is favorable [46,50,51,52]. In our experiments, the separation factor R_L_ was found to be in the range of 0.017–0.024 (Table 3). Thus, the results pointed out that the adsorption of all three studied metal cations (Cu^2+^, Cd^2+^, Pb^2+^) onto the oxidized wool fibers was favorable under the experimental conditions considered in this work.

**Figure 6:**
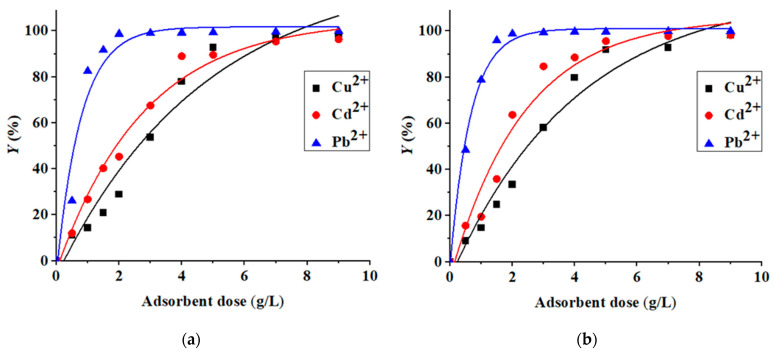
Removal efficiency of heavy metal cations (Cu^2+^, Cd^2+^, Pb^2+^) depending on the adsorbent dose: (**a**) removal efficiencies determined at 300 K, and (**b**) removal efficiencies determined at 323 K.

In addition, the removal efficiency (Y, %) of metal cations from aqueous solutions by adsorption onto the oxidized wool fibers is shown in Figure 6. Hence, the influence of the adsorbent dose on the removal efficiency was recorded for both temperature levels, i.e., 300 K (Figure 6a) and 323 K (Figure 6b). As one can see from Figure 6, the increment of the adsorbent dosage resulted in an increase in the removal efficiency. However, this increase tends to approach a horizontal asymptote; this effect is more evident for the removal of Pb^2+^ cations. The highest removal efficiency of metal ions was found to be 96.80 ± 1.69% (for Cu^2+^); 97.13 ± 1.09% (for Cd^2+^), and 99.70 ± 0.20% (for Pb^2+^).

Likewise, a comparative analysis was performed in order to compare the performances of our adsorbent with those registered for other keratin-based materials reported in the literature (Table 4). As one may see from Table 4, the adsorption performance of metal cations onto the oxidized wool fibers (obtained in this work) is intermediate if compared to other existing wool-based materials. However, the obtaining method of the oxidized wool reported in this work is simple and economically viable, enabling the production of a low-cost and environmental-friendly adsorbent.

#### 2.2.3. Thermodynamic Adsorption Parameters

To comprehend in more detail the nature of adsorption, one should calculate the basic thermodynamic parameters such as the Gibbs free energy, enthalpy, and entropy [50]. In order to estimate these thermodynamic parameters for the adsorption process, one should consider the equilibrium constants at least for two distinct temperature levels. The thermodynamic parameters of adsorption can be estimated by using the Equations (7)–(9) [46,50,51]:

(7) $$\Delta G=-R_{g}T\;\ln(K_{d})\;,$$

(8) $$\;\ln(K_{d})=\frac{\Delta S}{R_{g}}-\frac{\Delta H}{R_{g}T}\;,$$

(9) ΔG=ΔH−TΔS ,

where ΔG (kJ/mol), ΔH (kJ/mol), and ΔS (J/(K∙mol)) indicate the changes in Gibbs free energy, enthalpy and entropy of the adsorption process, respectively; T—the absolute temperature (K); R_g_—the universal gas constant (R_g_ = 8.314 J/(K∙mol)), and K_d_ is the equilibrium constant of adsorption. Note that, in this calculation, the equilibrium constant of adsorption was approximated with the Langmuir parameter (K_d_~K_L_) after converting the latter one (K_L_) into (L/mol) units.

On the basis of the above equations (Equations (7)–(9)), the values of the thermodynamic parameters were calculated and reported in Table 5. As given in Table 5, the positive values of the enthalpy (∆H) for Cd^2+^ and Pb^2+^ removal indicate endothermic processes, the adsorption of these cations being favored by the temperature increase. For Cu^2+^ removal, the negative value of the enthalpy suggested an exothermic process, meaning that a certain amount of heat was released during the adsorption of copper cations onto the surface of the oxidized wool. These different values of the enthalpies of adsorption (∆H) for the cations under consideration are in agreement with the experimental data at equilibrium reported in Figure 5.

The positive values of the entropy (∆S) summarized in Table 5 indicate an increase in the degree of freedom or disorder of the adsorbed species (metal cations) at the solid/solution interface. However, the entropy variation was smaller for Cu^2+^ removal, suggesting that this system was more stable from a thermodynamic standpoint and reached the equilibrium more rapidly as compared to the processes of Pb^2+^ and Cd^2+^ removal. The negative values of Gibbs free energy (∆G) indicate the spontaneity of the investigated adsorption processes aiming to remove Cd^2+^, Cu^2+^ and Pb^2+^ cations from aqueous solutions. In an approximation, the ranges of Gibbs free energy ∆G of (0 to −20 kJ/mol), (−20 to −80 kJ/mol), and (−80 to −400 kJ/mol) might be associated with physical adsorption, ion exchange, and chemisorption, respectively [59]. In this study, the values of ∆G are found between −33.74 and −28.59 kJ/mol suggesting the predominance of the electrostatic interactions (ion exchange) when retaining metal ions onto the oxidized wool fibers. In addition, we calculated the free energy of sorption (Es, kJ/mol) according to Dubinin–Radushkevich (D-R) isotherm. Hence, the free energy of sorption (Es, kJ/mol) was found to lay into the interval 14.78−28.98 kJ/mol. These data suggested that the adsorption mechanism was based on both ion-exchange and chemisorption. Thus, in the proposed Scheme 1c, the most probable route of the mechanism for cation retention is the path based on the electrostatic and coordinating interactions.

#### 2.2.4. Desorption Assay and Re-Use Tests

**Figure 7:**
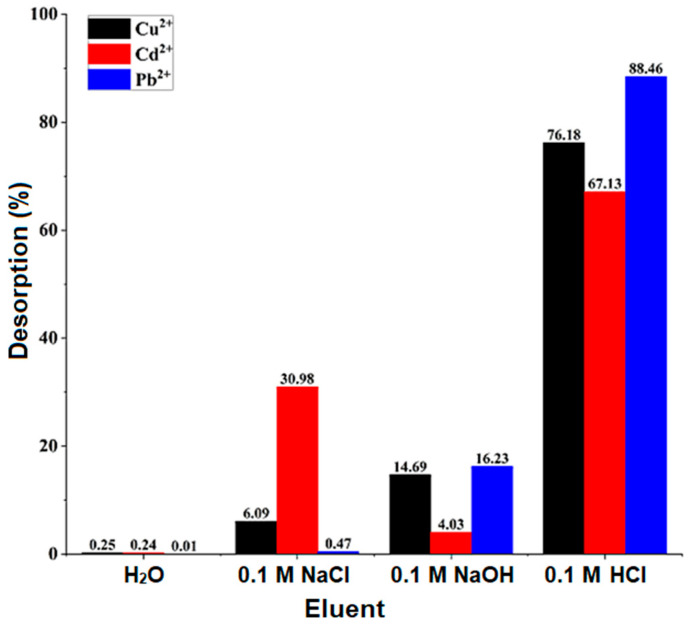
Desorption efficiency of metal ions from spent adsorbents (oxidized wool) in different eluent media (desorption time 1 h and working volume 15 mL).

Additionally, desorption experiments were carried out in batch mode by mixing the spent adsorbent samples (loaded with heavy metal cations) with different types of eluents (water or solutions of HCl, NaOH and NaCl). The results of desorption tests are shown in Figure 7. As seen from this diagram, the used liquid phases to desorb the cations from spent adsorbents can be ordered in a decreasing series of efficiencies as follows: HCl > NaOH ≈ NaCl > H_2_O. Maximum releases of heavy metal cations between 67 and 88% were obtained in 0.1 M HCl electrolyte solution (i.e., the best eluent). Thus, the adsorbed metal ions can be released in greater amounts at acidic pH, except for the cations linked in more stable mercaptide salts [24]. The reversibility of the wool–metal ion interactions is attributed to the competition between metal ions and hydrogen for the same active sites [21].

**Figure 8:**
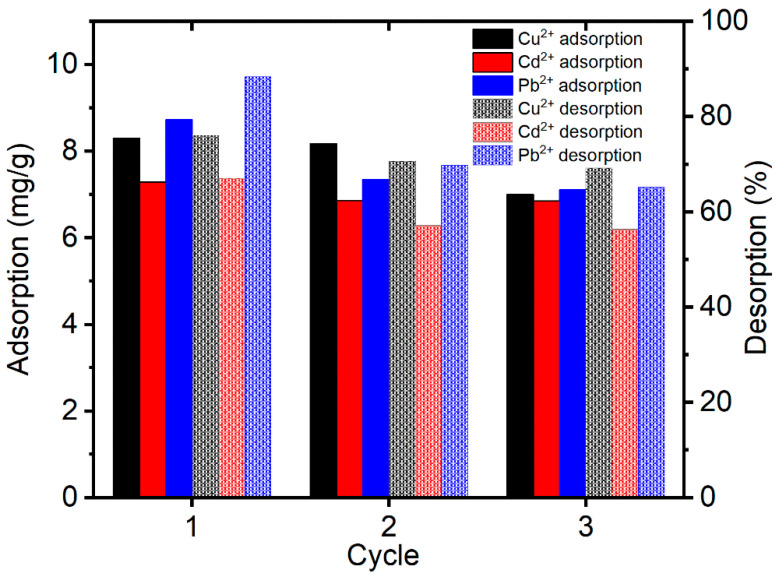
Consecutive adsorption–desorption cycles of heavy metal cations (Cu^2+^, Cd^2+^, Pb^2+^) onto the solid surface of oxidized wool fibers.

Considering the Figure 7 data, the sorbent samples recovered from HCl were subjected to two additional cycles of adsorption–desorption, following the same protocol as for the initial tests. The obtained results are presented in Figure 8. One may observe that, for all the studied heavy metal cations, the oxidized wool sorbent preserves the adsorption capacity almost unaffected for at least three consecutive cycles.

## 3. Materials and Methods

### 3.1. Materials

Chemical reagents of analytical grade were used as acquired, namely, ammonia 25% aqueous solution (NH_4_OH) from Chemical Company (Iasi, Romania), hydrogen peroxide 3%, solution (H_2_O_2_) from ChimReactiv (Bucharest, Romania), copper sulphate pentahydrate (CuSO_4_∙5H_2_O), cadmium nitrate tetrahydrate (Cd(NO_3_)_2_∙4H_2_O) and lead nitrate (Pb(NO_3_)_2_) from Merck Chemical (Saint Louis, MO, USA). Stock aqueous solutions of copper sulphate pentahydrate, cadmium nitrate tetrahydrate and lead nitrate were prepared at concentrations of 1 g/L using bi-distilled water.

The raw wool of very coarse grade (fiber diameter, 66.0 ± 6.7 μm) was supplied by a regional sheep farm (Todiresti, Vaslui, Romania). After removing solid macroscopic impurities, the raw wool was washed several times in tap water tempered at 45 °C to remove the lanoline, followed by rinsing with distilled water. Then, the wool fibers were dried in a laboratory oven at 35 °C for 24 h and stored for further use.

### 3.2. Oxidized Wool-Synthesis Protocol

The chemically modified wool was prepared by means of an oxidation process. To this end, a wool sample (raw fibers) was immersed in an alkaline solution, where oxidation was performed in the presence of hydrogen peroxide. The experimental protocol is detailed as follows: to a Berzelius beaker containing 2 L of distilled water, 256 mL of NH_4_OH (25%), and 400 mL of H_2_O_2_ (3%), was added 20 g of wool fibers. The mixture was left for 1 h at room temperature to ensure complete contact between solid and liquid phases. Then, the wool-containing solution was heated to about 95 °C for 1 h, under mechanical stirring. Than the oxidized wool fibers were removed from the reaction medium, washed several times with distilled water and twice with absolute ethanol, dried at room temperature for 24 h, and stored in a desiccator for further studies.

### 3.3. Characterization Techniques

The concentrations of the heavy metal cations in the solutions of the adsorption/desorption experiments were determined by atomic absorption spectrometry (AAS) using the ContrAA 800D, Analytik Jena spectrometer (Jena, Germany).

Fourier-transform infrared (FTIR) spectra of raw and modified materials were recorded by using a Bruker Vertex 70 model FTIR spectrometer (Ettlingen, Germany) in 4000–400 cm^−1^ wavenumber interval on KBr pellets. 

The fibrous morphology and the qualitative composition of the wool-based materials were examined by scanning electron microscopy (SEM) using an (ESCM) Quanta 200 device equipped with an energy-dispersive X-Ray analysis (EDX) module (Brno, Czech Republic).

### 3.4. Adsorption Assays for the Retention of Metal Cations

The adsorption of metal cations was carried out using a Biosan ES-20/60 incubator (Riga, Latvia) equipped with an orbital shaker and a temperature control system. The adsorption kinetics was followed on both single-component systems (i.e., retention of each metal cation separately) and a multi-component system (i.e., competitive retention of three metal cations simultaneously).

The kinetic experiments were performed in batch mode, at a temperature T = 300 K (27 °C) and pH = 5.0 ± 0.1. Thus, in an Erlenmeyer flask containing 50 mL of metal cation solution, 0.25 g of wool fibers were introduced under stirring (150 rpm). The initial concentration of heavy metal cations was fixed at 50 mg/L for the single-solute systems and 20 mg/L for each metal ion in the competitive system (i.e., 60 mg/L summing all three metal ions).

The adsorption isotherms were obtained in batch at various ratios between pollutant concentration and adsorbent dosage. In this respect, initial concentrations of metal cations were fixed at 50 mg/L, while the amount of the oxidized wool ranged from 0.5 to 9.0 g/L. The adsorption isotherms were recorded at two distinct temperatures of 300 K (27 °C) and 323 K (50 °C). The samples were stirred for 4 h at 150 rpm to reach equilibrium.

In all adsorption experiments, aliquots were taken from the liquid phase at different contact times and after reaching the equilibrium, the content of the heavy metal cations was determined by AAS. The adsorption kinetics was followed by using Equation (10):

(10) $$q_{t}=\frac{\left(C_{0}-C_{t}\right)V}{m\cdot 1000}\;,$$

where q_t_ (mg/g) is the amount of heavy metal adsorbed at time t that takes the maximum value (q_e_) at adsorption equilibrium (adsorption capacity of the wool); C_0_ and C_t_ (mg/L) are the metal concentrations of the initial solution and of the solution after time t of contact with wool fibers, respectively; V (mL) is the volume of the solution; m (g) represents the weight of the dried wool sorbent. In addition, the removal efficiency, Y (%), was calculated by means of Equation (11):

(11) $$Y=\frac{C_{0}-C_{e}}{C_{0}}\cdot 100\;,$$

where C_e_ is the residual metal ion concentration at equilibrium.

### 3.5. Desorption Assays of Metal Cations from Oxidized Wool Fibers and Re-Use Tests

Desorption studies were performed in distilled water and different eluents (HCl, NaOH and NaCl solutions of 0.1 M concentrations). Around 0.25 g of oxidized wool sorbent samples, previously exposed to the standard solutions of metal cations (according to the methodology described in Section 3.4 for kinetic experiments), were extracted, dried, and introduced into Erlenmeyer flasks containing 15 mL of each eluent. Desorption tests were performed at a temperature of 27 °C, under stirring (150 rpm), by using a Biosan ES-20/60 incubator, for 1 h. The metal ion concentrations in the desorption solutions were determined by AAS on a ContrAA 800D spectrometer. The desorption efficiency (%) was calculated as a ratio between the amount of metal ion desorbed and the amount of metal ion adsorbed (multiplied by 100) [60,61,62]. The oxidized wool samples recovered after desorption studies using HCl were evaluated for their re-use capacities for two supplementary cycles. Note that after recovering from acid solution, the materials were rinsed with NaOH 0.1 M to neutralize the HCl traces, and finally were washed thoroughly with distilled water.

## 4. Conclusions

In this study, we proposed a simple and efficient method for oxidizing wool fibers to obtain a modified keratin-based material with good adsorption properties for the removal of heavy metal ions (Cu^2+^, Cd^2+^, and Pb^2+^) from wastewater.

Kinetic data indicated that the adsorption equilibrium of metal ions onto oxidized wool fibers was attained at a contact time greater than one hour. The experimental observations were fitted to pseudo-first-order (PFO) and pseudo-second-order (PSO) kinetic models, for both single-component and competitive systems. In the case of the multi-component system (competitive) the results disclosed a higher selectivity for Cd^2+^ ions and a lower selectivity for Pb^2+^ ions. The reason for this might be attributed to the fact that the heavier ions (Pb^2+^) can diffuse more slowly toward the adsorbent surface than the lighter ones (Cd^2+^ and Cu^2+^).

The Langmuir and Freundlich models of equilibrium were applied to explore the adsorption isotherms at the two temperatures 300 K and 323 K. The Langmuir isotherm model showed better predictions than the Freundlich model for the studied systems (oxidized wool/heavy metal ions). Thus, the Langmuir equation better accounted for the adsorption of metal ions onto the oxidized wool fibers. At 300 K, the maximum adsorption capacities of oxidized wool fibers for the retention of Cu^2+^, Cd^2+^, and Pb^2+^ ions were equal to 9.41, 10.42, and 30.71 mg/g, respectively, more than double or triple times higher than the values corresponding to the retention of the same cations on pristine wool fibers. A higher temperature (323 K) resulted in somewhat greater adsorption capacities. Likewise, the removal efficiency of heavy metal ions from aqueous solutions by using the oxidized wool was about 96.80–99.70%, depending on the type of metal ion (Cu^2+^, Cd^2+^, or Pb^2+^). The experimental data suggested that the adsorption of Pb^2+^ and Cd^2+^ ions were favored by the increment in temperature, whereas the adsorption of Cu^2+^ ions was disfavored. 

According to thermodynamic calculations, the adsorption of Pb^2+^ and Cd^2+^ ions was accompanied by an endothermic effect, while the adsorption of Cu^2+^ ions by an exothermic effect. The negative values of the Gibbs free energy (∆G), ranging from −33.74 to −28.59 kJ/mol, suggested spontaneous adsorption, where the electrostatic interactions (ion exchange phenomena) might play a dominant role.

Desorption assays revealed the maximum releases (67–88%) of heavy metal ions from spent adsorbents in 0.1 M HCl electrolyte solution (the best eluent).

## Figures and Tables

**Table 1:** One-component adsorption kinetics: Kinetic models and parameters for individual adsorption of Cu^2+^, Cd^2+^ and Pb^2+^ cations onto the oxidized wool fibers; experimental conditions: T = 300 K, adsorbent dose = 5 g/L, C_0,i_ = 50 mg/L, and pH 5.0 ± 0.2.

Cation	q_e_^(obs) 1^	PFO Model			PSO Model		
		k_1_	q_e_(mg/g)	$\chi_{v}^{2}$	k_2_	q_e_ (mg/g)	$\chi_{v}^{2}$
Cu^2+^	8.30	0.091	7.971	0.308	0.015	8.604	0.053
Cd^2+^	7.29	0.128	7.280	0.069	0.023	7.804	0.311
Pb^2+^	8.73	0.673	8.495	0.032	0.174	8.688	0.029

^1^ q_e_^(obs)^ is the observed experimental value of absorption capacity (mg/g).

**Table 2:** Multi-component (competitive) adsorption kinetics: kinetic models and parameters for simultaneous adsorption of Cu^2+^, Cd^2+^ and Pb^2+^ cations onto the oxidized wool fibers; experimental conditions: T = 300 K, adsorbent dose = 5 g/L, ΣC_0,i_ = 60 mg/L, and pH 5.0 ± 0.2.

Cation	q_e_^(obs) 1^	PFO Model			PSO Model		
		k_1_	q_e_ (mg/g)	$\chi_{v}^{2}$	k_2_	q_e_(mg/g)	$\chi_{v}^{2}$
Cu^2+^	5.06	0.667	4.929	0.009	0.299	5.040	0.013
Cd^2+^	5.65	0.396	5.446	0.011	0.128	5.638	0.023
Pb^2+^	3.84	1.372	3.721	0.008	1.403	3.759	0.004

^1^ q_e_^(obs)^ is the observed experimental value of absorption capacity (mg/g).

**Table 3:** Isotherm models and parameters for adsorption of Cu^2+^, Cd^2+^ and Pb^2+^cations onto the oxidized wool fibers; experimental conditions: adsorbent dose = 0.5–9.0 g/L; C_0_ = 50 mg/L; t = 240 min, pH 5.0 ± 0.2.

Cation	T (K)	Freundlich Isotherm			Langmuir Isotherm			
		K_F_ (mg/g)(L/g)^1/n^	n_F_	$\chi_{v}^{2}$	q_m_ (mg/g)	K_L_ (L/mg)	R_L_	$\chi_{v}^{2}$
Cu^2+^	300	7.469	16.610	0.135	9.414	2.295	0.017	0.017
	323	6.733	9.354	0.639	10.062	1.218	0.018	0.167
Cd^2+^	300	4.592	4.391	0.839	10.423	0.457	0.024	0.239
	323	5.562	4.636	1.058	11.798	0.663	0.023	0.240
Pb^2+^	300	15.709	4.493	62.393	30.709	1.686	0.022	51.293
	323	19.859	4.541	79.586	37.849	2.815	0.021	66.340

**Table 4:** Comparison of the adsorption capacities of keratin-based materials for retention of heavy metal ions.

Adsorbent Material	Heavy Metal Ion	Adsorption Capacity (mg/g)	Adsorption Efficiency (%)	Ref.
Wool fibers (Erdenet city, Mongolia)	Cu^2+^	7.15	-	[18]
	Cd^2+^	6.26	-	
	Pb^2+^	4.30	-	
Wool fibers (“Askania-Nova”, Ukraine)	Cd^2+^	-	15.5	[53]
	Pb^2+^	-	28.0	
Wool powder	Cu^2+^	122.10	-	[54]
Keratin	Cu^2+^	0.55	-	[55]
	Cd^2+^	0.73	-	
Keratin	Pb^2+^	9.29	-	[56]
Keratin/Polyamide 6 (50/50)	Cu^2+^	6.19	-	[57]
Wool keratin/silk fibroin	Cu^2+^	2.90	-	[58]
Wool fibers treated with Na_2_S	Cu^2+^	26.04	-	[18]
	Cd^2+^	37.35	-	
	Pb^2+^	43.72	-	
Pristine wool fibers	Cu^2+^	2.49	-	This work
	Cd^2+^	3.87	-	
	Pb^2+^	8.66	-	
Oxidized wool fibers	Cu^2+^	9.41	96.80	This work
	Cd^2+^	10.42	97.13	
	Pb^2+^	30.71	99.70	

**Table 5:** Thermodynamic parameters calculated for the adsorption processes of Cu^2+^, Cd^2+^ and Pb^2+^ cations onto the oxidized wool fibers.

Cation	∆G (kJ/mol)	∆H (kJ/mol)	∆S (J/mol·K)
Cu^2+^	−29.94	−22.20	24.85
Cd^2+^	−28.59	12.97	133.44
Pb^2+^	−33.74	17.95	165.96

## Data Availability

Not applicable.
